# Comparison of Cochlear Microphonics Magnitude with Broad and Narrow Band Stimuli in Healthy Adult Wistar Rats

**Published:** 2018

**Authors:** FATEMEH HEIDARI PHD, AKRAM POURBAKHT, SEYED KAMRAN KAMRAVA, MOHAMMAD KAMALI, ABBAS YOUSEFI

**Affiliations:** 1 Department of Audiology, School of Rehabilitation Sciences, IranUniversity of Medical Sciences,Tehran, Iran .; 2 Department of Audiology, Schoolof Rehabilitation, Shahid BeheshtiUniversity of Medical Sciences,Tehran, Iran.; 3 ENT-Head & Neck Research Center, Hazrate Rasoul AkramHospital, Iran University of Medical Sciences, Tehran, Iran; 4 Department of Basic Sciences,in Rehabilitation, School of Rehabilitation Sciences, Iran University of Medical Sciences,Tehran, Iran; 5 Department of Medical Physics and Biomedical Engineering, Schoolof Medicine, Tehran University of Medical Sciences, Tehran, Iran.; 6 Department of Audiology, School of Rehabilitation, Tehran Universityof Medical Sciences, Tehran, Iran

**Keywords:** Cochlear microphonic potential, Auditory neuropathy, Electrocochleography, Rats, Evoked Potential

## Abstract

**Objective:**

Cochlear microphonic (CM) is a cochlear AC electric field, recorded within, around, and remote from its sources. Nowadays it can contribute to the differential diagnosis of different auditory pathologies such as auditory neuropathy spectrum disorder (ANSD). This study compared CM waveforms (CMWs) and amplitudes with broad and narrow band stimuli in 25 healthy male young adults Wistar rats.

**Materials & Methods:**

This experimental study was accomplished in the School of Rehabilitation Sciences of Iran University of Medical Sciences, Tehran, Iran (April, 2016). Using an extratympanic technique in ECochG (Electrocochleography) recording, CMWs in response to click and tonal stimuli with different octave frequencies were recorded at a high intensity level in subjects. The CMW amplitudes were calculated by a graphical user interface (GUI) designed in MATLAB.

**Results:**

The CMW magnitude increased upon an increase in bandwidth stimulation. CM amplitude with click stimulation was larger than tonal stimuli. Across tonal stimuli, the CMW amplitudes at lower frequency tones were larger than those at higher frequency tones. Those findings were statistically significant (*P*<0.001).

**Conclusion:**

CMW amplitude with click as broadband stimulus was larger than those with tone bursts as narrowband stimulation. Click stimulation due to the width of spectral involves greater regions of cochlear partition. Therefore, CMW most likely is a reflection of spatial summation of voltage drops generated by hair cell groups in response to acoustic stimulation. In order to production nature of CM potentials as well as their very small magnitudes especially with tonal stimuli, thus, we recommend using click stimulation for CM potential recording.

## Introduction

The cochlear microphonic (CM) is an alternative current (AC) voltage and one of the auditory receptor potentials (1, 2). Its generators are mechanoelectrical transduction currents through the population of hair cells (mainly outer hair cells (OHCs)) and the driving force of endocochlear potential (EP) evoked in response to auditory stimuli. It is elicited by basilar membrane (BM) displacement and stereocilia deflection (1, 3, 4). On the other hand, CM represents extracellular voltage alterations in OHCs dominated basally and possibly some receptor currents of Inner hair cells (IHCs) (5). CM is a preneural, sustained response and follows the waveform of the acoustic stimuli (6). This product of cochlear hair cells can be recorded in humans and experimental animals at several recording sites (7). Although more than 80 yr have passed since the discovery of CM, its application in clinical settings was restricted due to limitations such as the use of invasive transtympanic electrode array and electromagnetic interaction (8-10). 

Currently, CM recording has attracted new interest because it has an important role in the diagnosis of auditory neuropathy spectrum disorder (ANSD) (11-14). This disorder is characterized by absent or severely abnormal ABR with OAE and/or CM preservation, which are indicators of OHCs integrity. The incidence of ANSD is higher in infants especially treated in the neonatal intensive unit care (NICU) (12-15). 

The clinical application of OAE has been widespread, promoted particularly by the introduction of universal neonatal audiological screening programs. OAE is an easy, quick, noninvasive, and objective test, which measures cochlear amplification function (16). Despite the advantages of OAE, there are some limitations including restricted measurement and high levels of artifacts due to background noise involving acoustic and physiologic noise (10). The ABR recording is the most common test for threshold estimation, especially for pediatric and neurotologic purposes for which OAE cannot be used. The other limitation of OAE is vulnerability to middle ear (ME) disease, such as effusion otitis media, which has a high prevalence in infants and children (15).

In contrast to OAE, CM is an electrical signal and is not influenced by acoustic noise. It can be measured simultaneously with ABR recording, therefore saving money and time (10). The measurable frequency range in CM is greater than that of OAE and the former is resistant to ME pathologies. In addition, some studies reported greater stability of CM than of OAE in ANSD; OAE disappeared during the time course of the disorder but CM even showed a high amplitude and long duration in some patients (12, 14). Similar generators have been indicated for both OAE and CM; however, the cochlear mechanisms underlying these responses are different. For instance, their dual behavior against crossed olivocochlear bundle function can confirm this claim. Stimulation of an efferent auditory system increases CM amplitude whereas the magnitude of OAE decreases (14). Moreover, in a prestin knockout mouse model study, CM was similar to that of the wild-type mice. CM was not influenced by the cochlear amplifier (6). Hence, application of CM and OAE provides more information about the functional nature of the cochlea and gives an extended view of its analysis. 

Unlike OAE, there is lack of normative variation of CM with click and especially with tonal stimuli at different frequencies in Wistar rats, commonly utilized in hearing research. A study indicated the amplitude of CM with tonal stimuli shifted according to frequency (17), however, the associated effects of stimulation bandwidth and frequency on CM magnitude have not yet been investigated. 

We aimed to compare CM amplitude and waveform with click and tonal stimuli at octave frequencies of 2, 4, 8 and 16 kHz in healthy male young adult Wistar rats.

## Materials and Methods

**Animals**

The current study was conducted in the school of Rehabilitation Sciences of Iran University of Medical Sciences (Tehran, Iran) on April, 2016. Twenty-five healthy, male, young adult Wistar rats weighting 200–250 gr were used as subjects. The rats were purchased from the Center of Experimental and Comparative Studies of Iran University of Medical Sciences (Tehran, Iran) and housed with free access to water and food in their cages. The rats were maintained at a temperature of 22–24 °C, with 50% humidity, and on a 12/12-h light/dark cycle. 

The study was approved by the Ethics Committee of Iran University of Medical Sciences [No. 93.d.105.6113] and was conducted in accordance with the regulations for the use and care of animals in research.

**CM Recording**

Stimulation delivery and CM recording were performed in a sound-attenuating shielded booth. Electromagnetic shielding and grounding of the electrodes cables were used for reducing stimulus artifacts (9). CM recording was conducted for all Wistar rats using the Biologic Navigator pro system (Natus, USA). The custom tonal stimuli were in the WAV format and consisted of a 5-ms tone burst at frequencies of 2, 4, 8 and 16 kHz. Click stimulation of 0.1-µsec duration was also used. Before the experiments, the output of the transducer was measured in SPL for all stimuli with a sound level meter (Bruel & Kjaer 2250, Denmark). Prior to eliciting CM, anesthesia was induced using a combination of ketamine (80 mg/kg) and xylazine (5 mg/kg) intraperitoneally.

**Fig 1:**
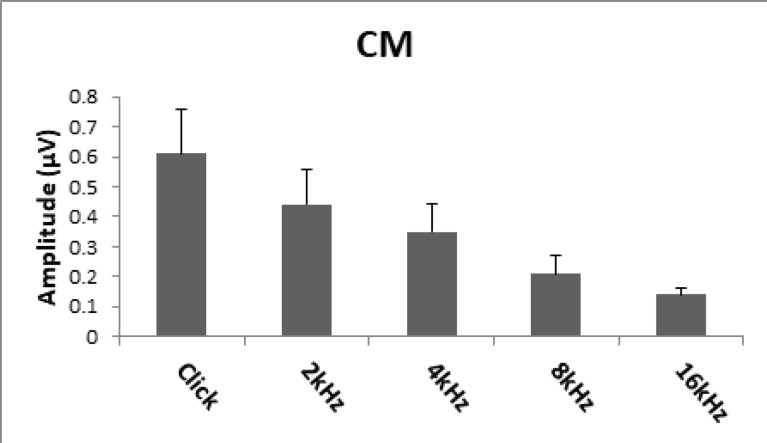
Mean of maximum amplitude of CM

**Fig 2:**
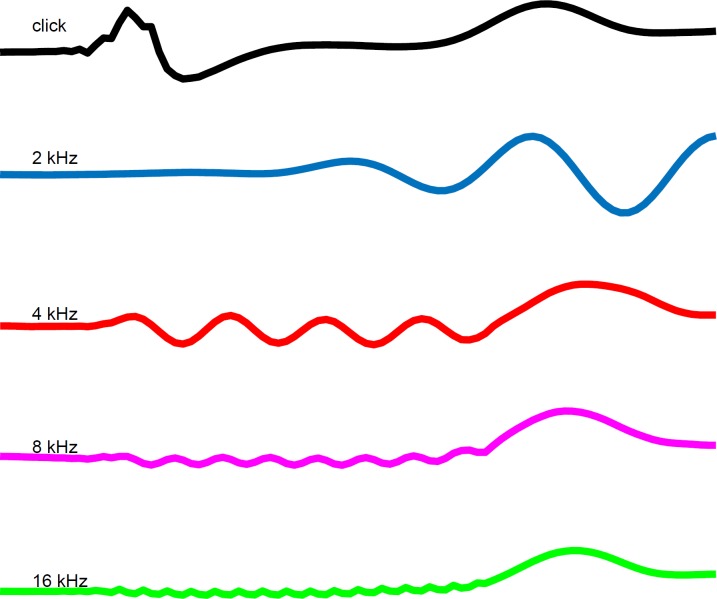
CMWs with broad and narrow band stimuli

Three subcutaneous needle electrodes were placed at vertex (non-inverting), under the right (inverting) and the left (ground) ears as in an ABR electrode array (18). If the electrode impedance at three sites was lower than 5 kΩ and the inter-electrode impedance was <2 kΩ, then calibrated stimuli were delivered by loudspeaker located 5 cm from the right ear at 80 dB SPL, with the stimuli and acquisition parameters including: 7.1/sec as the repetition rate, a 5.33-ms time window with 1-ms prestimulation, sampling of 256 points, amplified×100000, a bandpass filter of 100–1500 Hz, and an average of 1000 waveforms. To confirm appropriate recording methods and true CM recordings, the following approaches were used: phase CMWs inversed with alterations in stimulus single polarity involving rarefaction and condensation, alternating polarity eliminated CMWs (7, 15), and they followed the stimulation duration and frequency (15, 18). CMWs that met these criteria were accepted as true.

At each stimulus set, two records were obtained and stored for offline analysis. For signal processing, the raw CM signals were taken from the Biologic Evoked Potential system in ASCII format and transferred to a personal computer running Mathworks MATLAB software (Version 8.1, R2013a). For analysis of CM waveform and calculation of CM amplitude, we designed a graphical user interface (GUI). Analysis of CM magnitude was based on baseline-to-peak amplitude. CM amplitude (µV) was defined as the difference between the baseline voltage and the largest positive peak of the CM waveform with rarefaction polarity within initial 1-ms after stimulation onset between 40 to 80 points to prevent stimulus artifact and compound action potentials intervention.

**Statistical Analysis**

Mean and standard deviation (SD) is expressed for all data. One-way ANOVA was performed for comparison of the amplitude of CM with different stimuli and a final post hoc analysis was employed using Scheffe’s test. *P*<0.05 was considered statistically significant.

## Results

CM amplitude data indicated that there was a direct relationship between bandwidth of stimuli (click versus tonal stimuli) and CM magnitude. Figure 1 shows that by increasing the bandwidth, the amplitude of CM increased. That is, the CM amplitude of click was greater than that of tonal stimuli. Across four tonal stimuli, there was an inverse relationship between CM amplitude and frequency. As the stimulation frequency increased, the magnitude of CM decreased. 

Figure 1 shows mean values of maximum amplitudes of CM (in µV) at five stimuli (click versus tonal stimuli) in 25 male, young adult Wistar rats. There was a direct relationship between CM amplitude and bandwidth (*P*<0.001). In addition, in narrowband stimuli, lower frequencies had greater CM amplitude than higher frequencies did (*P*< 0.001).

One-way ANOVA revealed that the mean difference in CM magnitudes across five stimuli was statistically significant (*P*<0.001). Furthermore, Scheffe’s post-hoc test confirmed that there was a significant discrepancy (*P*<0.001) between the mean CM amplitudes of click (0.61±0.16 µV) versus tonal stimuli as well as across the tonal stimuli. There was a significant difference (*P*< 0.001) between the CM amplitude at 16 kHz (0.14±0.02µV) and 8 kHz (0.21±0.06µV) with 4 kHz (0.35±0.09µV) and 2 kHz (0.44±0.12µV).

In addition, Figure 2 presents the CMWs for all tested stimuli in one subject. As was observed, CMWs follow stimulation frequency, polarity, and waveform.

Five traces from top to bottom represent click, 2000, 4000, 8000, and 16000 Hz waveforms, respectively, in response to stimulus intensity level at 80 dB SPL with rarefaction polarity. 

## Discussion

The aim of the current study was to compare CM amplitude with different stimuli (Click as broadband stimulus and tone bursts as narrowband stimuli at different octave frequencies). Click has been known as a short time stimulus which spreads over a wide range of frequencies, whereas the time of tonal stimulus is longer, but has a narrow frequency spread (15). Our question was how does CM behave in terms of its amplitude and waveform in response to various stimuli at a constant high-intensity level (80 dB SPL) in healthy young adult Wistar rats? Rats are attractive as auditory system models. The reasons for this are their availability and their similar genetic features as well as hearing characteristics to those of humans. Therefore, auditory results in a rat model can be extrapolated to human hearing (1).

The findings of the current study revealed that the CM amplitude with click stimulus was generally larger than that with tonal stimuli. Across four tonal stimuli, there was an inverse relationship between the CM amplitude and frequency. In other words, higher frequencies showed smaller CM amplitude than lower frequencies did, and this difference was statistically significant. Regarding the polarity and waveforms of stimulation, CMWs followed those stimulation features. Similarity among these stimulus properties and CMWs is a basic characteristic of CM potential. According to the nature of their generation and the physiologic mechanisms underlying CM and frequency tuning curve (FTC), there are some possible explanations for the findings of this study. 

In response to acoustic stimulus, movement is induced in the cochlear fluids, traveling waveforms and following BM displacement, travels from base to apex according to its graded stiffness. This traveling wave moves toward the helicotrema regardless of where the stimulus was applied. The wave is an extremely important component in the analysis of sound by the auditory system. The frequency of the stimulus influences the pattern and position of the wave (19, 20). 

When the traveling waveforms with delivering acoustic stimulus and its amplitude peaks at CF, fast phase changes occur around the peak of the traveling wave, thus large cancelation happens physiologically in hair cells in response to the CF. “Therefore, the CM is dominated by hair cell receptor currents generated by the more linear tails (non-amplified portion of hair cell tuning curve) of mechanical excitation patterns” (6). Tuning curves of the hair cells, which are similar to the BM tuning curve at different frequencies. Since, the region of tail portion at low frequencies is more extended than high frequencies, as well as low frequencies are analyzed in apical turn of cochlea, hence their traveling waves move a greater distance along the cochlear duct, and as a result, more OHCs are affected. Additionally, the volume of hair cells in an apical turn is greater than in a basal turn. Broadband click stimulation is not frequency-specific and its traveling wave is spread along the cochlear partition from base to apex and involves more hair cells than narrow band stimuli does. In fact, CM is a reflection of the spatial summation of hair cell receptor currents (4, 17). These physiologic properties can infer that greater numbers of hair cell groups contribute to CM production during broadband stimulation and with narrow band lower frequencies rather than with high frequencies, and this leads to larger CM amplitude for click stimulus and lower frequencies. The other hypothesis explaining these findings is the nature of the intrinsic low-pass filter of hair cells (8); when stimulation frequency increases, AC potential decreases despite the fact that DC potential increases. In addition, in vivo study in mice has shown a variety of stimulus intensity–CM magnitude functions for different frequencies (17). The saturation level for low frequency was higher than that of the high-frequency tones. That is, the amplitude of CM potential at low frequencies in mice grew with increasing stimulation level. The present study was conducted with a high-intensity level; therefore, low frequencies were able to increase the AC field more than high frequencies were.

Limited previous studies using animal models or human research reported that lower frequencies had larger CM amplitude than higher frequencies did (17, 21). However, to our knowledge, a comparison of CM amplitude according to click and tonal stimuli had not yet been conducted.

**In conclusion**, the CM amplitude was influenced by the bandwidth of the stimulation. CM amplitude with click was larger than tonal stimuli. In addition, across tonal stimuli with different octave frequencies, there was inverse relationship between CM amplitude and frequency. Since, click stimulus spreads over a wider range of frequencies, and traveling wavelength of low-frequency tonal stimuli are longer than high-frequency ones, this very small AC cochlear potential is a reflection of the spatial summation of hair cell groups according to traveling wave propagation along the cochlear partition. Thus, we observed greater amplitude of CMWs in click, low tonal, and high tonal stimuli, in descending order. Therefore, click instead of tonal stimuli results in lesser time and larger amplitude, and it is better to measure cochlear evoked potentials with click stimulation as a test approach in some special conditions such as ANSD.
